# Supplementary material for: Identification of photoperiod-induced specific miRNAs in the adrenal glands of Sunite sheep (Ovis aries)
Source: Front Vet Sci. 2022 Jul 22;9:888207. doi: 10.3389/fvets.2022.888207 (PMC9354845; doi:10.3389/fvets.2022.888207)
Supplement: Supplementary file 1 [file Data_Sheet_1.ZIP › Supplementary file/miRDeep2 file.zip!/miRDeep2 file/Know_Structure/LP42A3/oar-mir-29a.pdf]

miRBase precursor : oar-mir-29a
Total read count : 153464
oar-miR-29a read count : 153430
remaining reads : 34

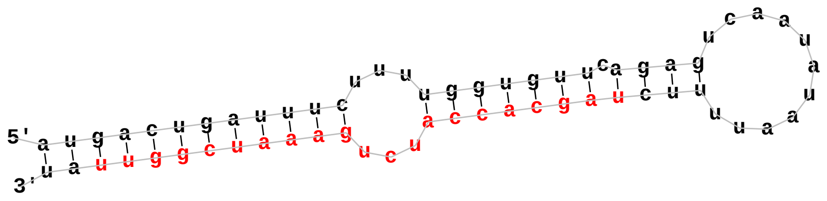

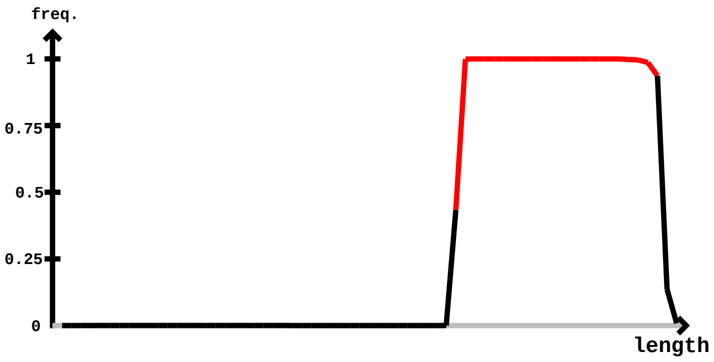

oar-miR-29a

```
5'- augacugauuucuuuugguguucagagucaauauaauuuucuagcaccaucugaaaucgguuau   -3'    exp
    ((((((((((((...(((((((.((((...........)))))))))))...))))))))))))    reads      mm        sample
    .ugacugauuucuuuugguguuc.........................................    14         0         seq
    ..gacugauuucuuuugguguuc.........................................    1          0         seq
    ...acugauuucuuuugguguuc.........................................    15         0         seq
    .....ugauuucuuuugguguuUaga......................................    1          1         seq
    ...........................ucaauauaauuuucuagcaccaucug...........    1          0         seq
    ......................................uucuagcaccaucugaaaucgguu..    2          0         seq
    .......................................ucuagcaccaucugaaaucgg....    6          0         seq
    .......................................ucuagcaccaucugaaaucggu...    15         0         seq
    .......................................ucuagcaccaucugaaaucgguu..    7          0         seq
    .......................................ucuagcaccauUugaaaucgguu..    2          1         seq
    ........................................cuagcaccaucugaaGu.......    1          1         seq
    ........................................cuagcaccaucugaaau.......    4          0         seq
    ........................................Auagcaccaucugaaau.......    1          1         seq
    ........................................cuagGaccaucugaaau.......    1          1         seq
    ........................................cuagcaccaucugaaaA.......    1          1         seq
    ........................................cuagcaccaucugaaauA......    3          1         seq
    ........................................cuagcaccaucugaaauc......    81         0         seq
    ........................................cuagcaccaCcugaaauc......    1          1         seq
    ........................................cuagcaccaucugaaaucg.....    66         0         seq
    ........................................cuagcaccaucugaaaucgg....    344        0         seq
    ........................................cuagcaccauUugaaaucggu...    26         1         seq
    ........................................cuagcaccaucugaaaucggu...    2656       0         seq
    ........................................cuagcaccauUugaaaucgguu..    222        1         seq
    ........................................cuagcaccaucugaaaucgguu..    61704      0         seq
    ........................................cuagcaccaucugaaaucgguua.    1297       0         seq
    ........................................cuagcaccauUugaaaucgguua.    3          1         seq
    ........................................cuagcaccaucugaaaucgguuau    55         0         seq
    ........................................cuagcaccauUugaaaucgguuau    1          1         seq
    .........................................uagcaccUucugaaauc......    1          1         seq
    .........................................uagcaccaucugaaauU......    18         1         seq
    .........................................uagcaccaucugaaauc......    193        0         seq
    .........................................uagcaccaucugaaauA......    8          1         seq
    .........................................uagcUccaucugaaauc......    1          1         seq
    .........................................Aagcaccaucugaaauc......    2          1         seq
    .........................................uagcaccaCcugaaauc......    1          1         seq
```

oar-miR-29a

```
augacugauuucuuuugguguucagagucaauauaauuuucuagcaccaucugaaaucgguuau
.........................................uagcacUaucugaaauc......   1      1    seq
.........................................uagcaccaucugaaaucg.....   192    0    seq
.........................................uagcaccauUugaaaucg.....   13     1    seq
.........................................uagcaccaucugaaaucgg....   1089   0    seq
.........................................uagcaccauUugaaaucgg....   61     1    seq
.........................................uagcaccauUugaaaucggu...   464    1    seq
.........................................uagcaccaucugaaaucggu...   4431   0    seq
.........................................uagcaccaucugaaaucgguu..   58692  0    seq
.........................................uagcaccauUugaaaucgguu..   2282   1    seq
.........................................uagcaccaucugaaaucgguua.   17440  0    seq
.........................................uagcaccauUugaaaucgguua.   728    1    seq
.........................................uagcaccaucugaaaucgguuau   1192   0    seq
.........................................uagcaccauUugaaaucgguuau   53     1    seq
..........................................agcaccaucugaaaucgg....   1      0    seq
..........................................agcaccaucugaaaucggu...   1      0    seq
..........................................agcaccauUugaaaucgguu..   4      1    seq
..........................................agcaccaucugaaaucgguu..   46     0    seq
..........................................agcaccaucugaaaucgguua.   15     0    seq
..........................................agcaccauUugaaaucgguua.   1      1    seq
...........................................gcaccaucugaaaucgguu..   1      0    seq
...........................................gcaccaucugaaaucgguua.   2      0    seq
```
